# Addressing Barriers to Care in Hepatocellular Carcinoma: Promoting Equity and Access

**DOI:** 10.7759/cureus.41893

**Published:** 2023-07-14

**Authors:** Sindhu Vikash, Fnu Vikash, Aarushi Sudan, Bisrat Adal, Donald Kotler

**Affiliations:** 1 Department of Internal Medicine, Jacobi Medical Center, Albert Einstein College of Medicine, Bronx, USA

**Keywords:** healthcare disparity, racial inequities, racial disparity, hbeag-negative, pre-core mutant, hepatocellular carcinoma, hepatitis b virus

## Abstract

Chronic hepatitis B virus (HBV) infection is the leading cause of hepatocellular carcinoma (HCC). Chronic viral hepatitis is projected to surpass the composite mortality rates of the human immunodeficiency virus (HIV), tuberculosis, and malaria by 2040. It can be attributed to several barriers to chronic HBV infection (CHBVI) surveillance that warrant urgent attention. Here, we report a case of a 40-year-old male with CHBVI who developed HCC and underwent partial hepatic resection. However, due to an interruption in insurance and medication regimen, the patient became the victim of healthcare disparity, which led to the progression of HCC and succumbed to widespread metastasis. This case highlights and discusses the healthcare disparity and critical value of continuity of care for patients with HBV infection to promote optimal patient outcomes.

## Introduction

The hepatitis B virus (HBV) epidemic is a leading public health concern despite the accessibility of highly effective vaccines [1]. Worldwide, almost 350 million people are chronically infected with HBV, which accounts for more than one million deaths due to progressive liver diseases like cirrhosis and hepatocellular carcinoma (HCC) [2]. Chronic HBV infection (CHBVI) progresses through four distinct phases - (i) immune tolerant, (ii) immune clearance (hepatitis B e antigen {HBeAg}-positive chronic hepatitis), (iii) non-replicative (inactive carrier state), and (iv) reactivation (HBeAg-negative chronic hepatitis) [3]. During the immune tolerant phase, there is the presence of HBeAg, high HBV deoxyribonucleic acid (DNA) levels, and normal aminotransferase with minimal or no inflammation on liver biopsy [3]. The immune clearance phase is characterized by HBeAg, high or fluctuating serum HBV DNA levels, persistent or intermittent elevation in serum aminotransferases, and active inflammation on liver biopsy. The subsequent non-replicative (inactive hepatitis B surface antigen {HBsAg} carrier state) is marked by the absence of HBeAg, presence of hepatitis B e antibody (HBeAb), low or undetectable serum HBV DNA, persistently normal aminotransferase levels, and minimal inflammation and fibrosis on liver biopsy. Finally, the reactivation phase (HBeAg-negative chronic hepatitis) is characterized by episodic negative HBeAg, positive anti-HBe, detectable HBV DNA, elevated aminotransferases, and continued necroinflammation leading to fibrosis. The American Association for the Study of Liver Diseases (AASLD) recommends regularly monitoring liver inflammation and viral activity in patients diagnosed with CHBVI [4]. On the contrary, several studies reported inadequate monitoring and treatment among patients with CHBVI in the United States (US) [5-7]. Based on Gilead Sciences estimation, Cohen et al. speculated that CHBVI was undertreated in the United States, and only approximately 50,000 of an estimated 1.4-2 million patients with CHBVI in 2010 were prescribed antiviral medications [8]. Here, we report and discuss a 40-year-old male with CHBVI who lost to follow-up due to insurance interruption, presented in the reactivation phase, and faced life-threatening consequences. Furthermore, this study highlights the critical need for continuity of care of patients with HBV infection to promote optimal patient outcomes.

## Case presentation

A 40-year-old male from Ghana with a past medical history of CHBVI who immigrated to the United States in 2012 presented to the emergency department (ED) with the chief complaint of the progressive right upper quadrant (RUQ) pain for one week. The pain was severe, progressive, sharp, and associated with nausea, vomiting, subjective fever, night sweats, generalized weakness, and dark urine for the past two weeks. The patient had no relevant family or occupational history but had a significant history of smoking (2.25 pack-years) and alcohol (two drinks/day for 10 years). Vital signs were within normal limits. Physical examination was remarkable for RUQ palpable mass and tenderness.

The patient was initially diagnosed with HBV infection in late 2018, and the hepatitis panel demonstrated reactive HBsAg and HBeAg. Hepatitis B virus-deoxyribonucleic acid polymerase chain reaction (HBV-DNA PCR) was 13,000,000 IU/mL, which climbed up to 85,450,998 IU/mL in three months. The source of acquiring infection was unclear despite the completion of the hepatitis B vaccine series before immigrating to the United States. At the time of diagnosis of HBV infection in 2018, liver ultrasonography (US) showed a hypoechoic mass in the inferior right lobe (2.1 × 1.5 × 2.1 cm) with associated hypervascularity. Subsequently, the patient was started on tenofovir alafenamide fumarate (VEMLIDY) 25 mg tablet daily in early 2019. Magnetic resonance imaging (MRI) during the same year showed a 2.5 cm hypervascular hepatic lesion compatible with Liver Imaging Reporting and Data System (LI-RADS) (LR-5), highly suggestive of HCC. Additionally, liver elastography showed that the patient had stage 2-3 fibrosis and was determined to have compensated cirrhosis (model for end-stage liver disease {MELD} score 7). Despite being offered a liver transplant, the patient declined it, as he firmly believed in his own ability to recover and instead opted for a hepatic wedge laparoscopic resection in 2019. Subsequently, he had an HBV viral load of less than 10 IU/mL and was adherent to VEMLIDY. However, he missed follow-up visits with his primary care physician and oncologist. Patient’s MRI of the abdomen in 2020 demonstrated new lesions in segments V and VI concerning for reoccurrence of HCC. Subsequently, the patient lost health insurance coverage due to changing jobs and stopped taking VEMLIDY in 2021.

The patient presented to our hospital in late 2022 with severe RUQ pain. Laboratory workup on presentation showed normal complete blood count (CBC), mild transaminitis, reactive HBsAg and HBeAb, non-reactive HBeAg and hepatitis B surface antibody (HBsAb), HBV-DNA PCR 43,500 IU/mL with mildly elevated carcinoembryonic antigen (CEA), and alpha-fetoprotein (AFP). The rest of the hepatitis A, C, D, and E panels and human immunodeficiency virus (HIV) serology were negative (Table 1).

**Table 1:** Laboratory test results at the time of admission. WBC: white blood cell; HGB: hemoglobin; PLT: platelet count; AST: aspartate aminotransferase; ALT: alanine aminotransferase; ALP: alkaline phosphatase; PT: prothrombin time; INR: international normalized ratio; HBsAg: hepatitis B surface antigen; HBsAb: hepatitis B surface antibody; HBeAg: hepatitis B e antigen; HBeAb: hepatitis B e antibody; HBV-DNA PCR: hepatitis B virus DNA polymerase chain reaction; HbcAb: hepatitis B core antibody; CEA: carcinoembryonic antigen; AFP: alpha-fetoprotein

Laboratory test	Result	Reference range
WBC	6.78/nL	3.90-10.60/nL
HGB	14 g/dL	13.5-17.5 g/dL
Platelet count	149/nL	150-440/nL
AST	64 U/L	<40 U/L
ALT	35 U/L	<40 U/L
ALP	75 U/L	<130 U/L
Total bilirubin	0.5 mg/dL	<1.2 mg/dL
Albumin	4 g/dL	3.5-5.5 g/dL
PT/INR	13.4/1.3	9.4-12.5 s/0.8-1.1 ratio
HBsAg	Reactive	-
HBsAb	Non-reactive	-
HBeAg	Non-reactive	-
HbeAb	Reactive	-
HBV-DNA PCR	43,500 IU/mL	-
HbcAb IgM	Non-reactive	-
HbcAb IgG	Reactive	-
Hepatitis A panel	Non-reactive	-
Hepatitis C panel	Non-reactive	-
Hepatitis E panel	Non-reactive	-
HIV test	Non-reactive	-
CEA	26.7 ng/mL	0.2-5.0 ng/mL
AFP	375 µg/L	0.0-9.0 µg/L

**Figure 1:**
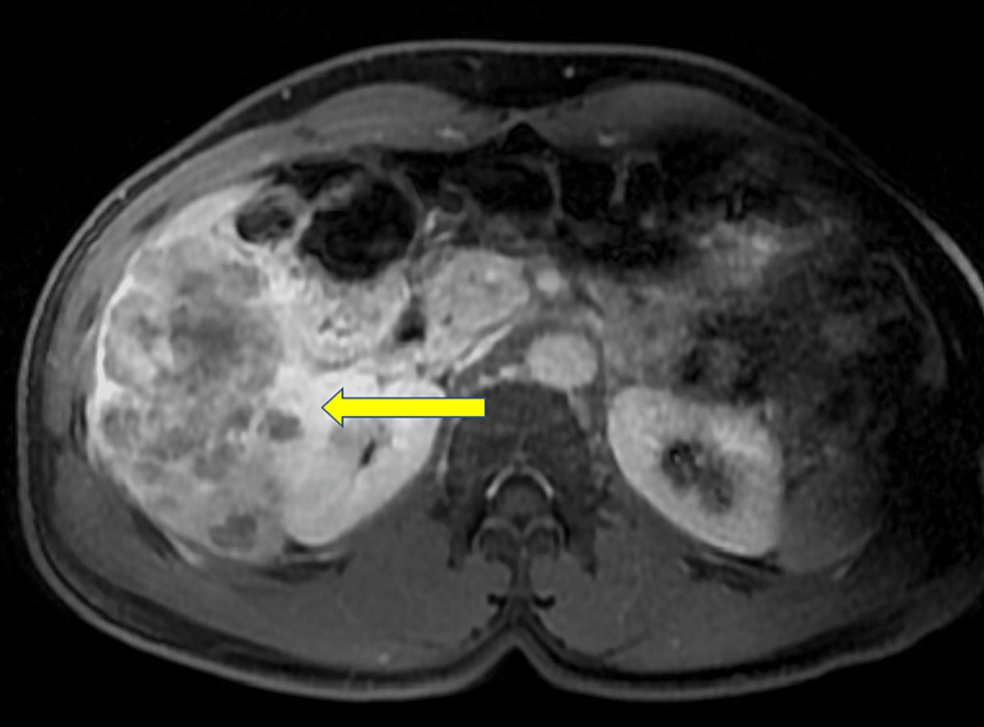
An axial MRI image with contrast showing a 9 cm conglomerate mass made up of multiple targetoid masses in the right liver lobe (arrow).

MRI of the abdomen showed a 9 cm conglomerate mass of multiple targetoid masses in the right liver lobe and a few sub-centimeter lesions in the left liver lobe (Figure 1). After successfully managing the patient's pain, he was discharged from the hospital on VEMLIDY, and an outpatient oncology follow-up was scheduled to get atezolizumab-bevacizumab chemotherapy. However, due to worsening RUQ pain patient was readmitted before getting outpatient chemotherapy. Workup was remarkable for worsening transaminitis, hyperbilirubinemia, hypoalbuminemia, and severe lactic acidosis. HBV-DNA PCR jumped to >1,000,000,000 IU/mL (Table 2).

**Table 2:** Laboratory test results on readmission. AST: aspartate aminotransferase; ALT: alanine aminotransferase; ALP: alkaline phosphatase; PT/INR: prothrombin time/international normalized ratio; HBV-DNA PCR: hepatitis B virus DNA polymerase chain reaction

Laboratory test	Result	Reference range
AST	1362 U/L	<40 U/L
ALT	838 U/L	<40 U/L
ALP	175 U/L	<130 U/L
Total bilirubin	20 mg/dL	<1.2 mg/dL
Direct bilirubin	1.4 mg/dL	0.1-0.3 mg/dL
Albumin	2.2 g/dL	3.5-5.5 g/dL
PT/INR	20.5/1.8	9.4-12.5 seconds/0.8-1.1 ratio
Lactic acid	7.9 mmol/L	0.6-1.4 mmol/L
HBV-DNA PCR	>1,000,000,000 IU/mL	-

**Figure 2:**
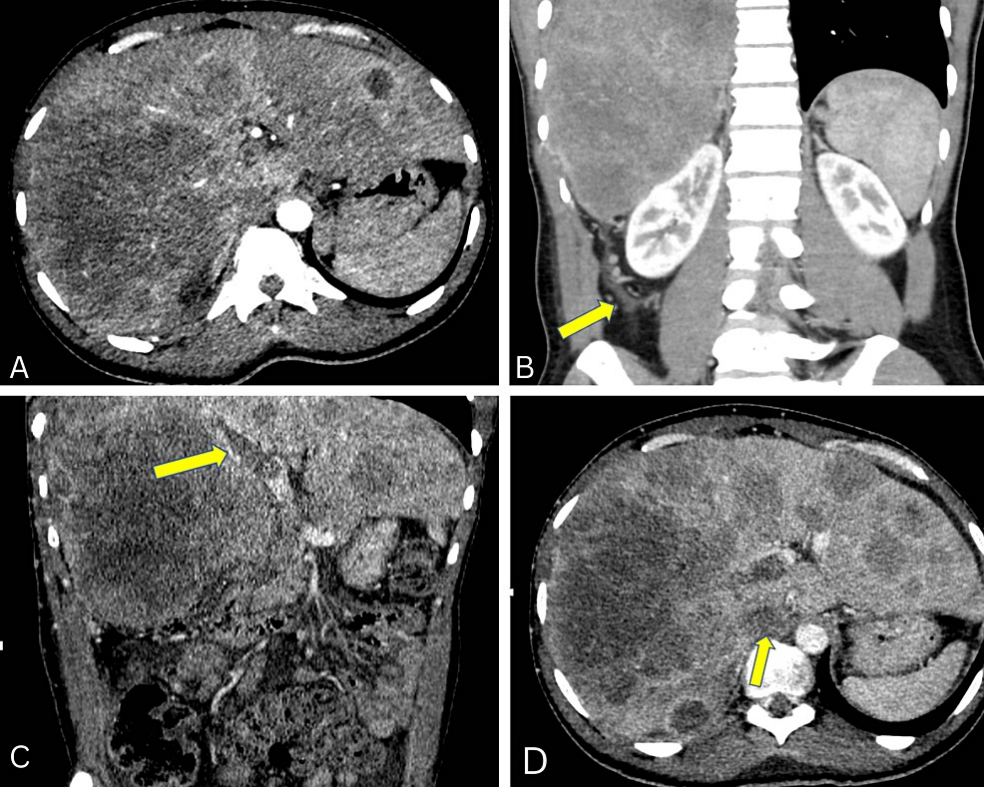
CT abdomen of the patient. (A) Axial section shows innumerable hepatic masses replacing the entire right hepatic lobe, involvement of greater than 50% of the left hepatic lobe, (B) coronal section shows omental metastasis (arrow), (C) coronal section shows portal vein tumor thrombus (arrow), and (D) axial section of CT scan showing intrahepatic IVC involvement (arrow). CT: computed tomography; IVC: inferior vena cava

The patient experienced frequent pain episodes in the hospital hence repeat CT abdomen demonstrated innumerable hepatic masses replacing the entire right hepatic lobe, involvement of greater than 50% of the left hepatic lobe (Figure 2A) with omental metastasis (Figure 2B), occluded right portal vein (Figure 2C) and right hepatic vein suggesting tumor thrombus extending into the intrahepatic inferior vena cava (Figure 2D).

**Figure 3:**
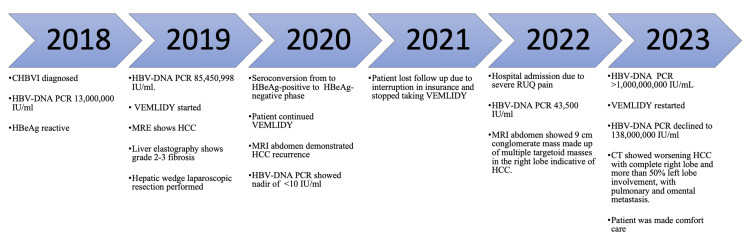
Timeline of significant events in the patient's disease course. CHBVI: chronic hepatitis B virus infection; HBV-DNA PCR: hepatitis B virus deoxyribonucleic acid polymerase chain reaction; HBeAg: hepatitis B e antigen; MRE: magnetic resonance elastography; HCC: hepatocellular carcinoma; MRI: magnetic resonance imaging; RUQ: right upper quadrant; CT: computed tomography

The patient was not a candidate for an atezolizumab-bevacizumab (chemotherapy) regimen due to worsening liver function. Moreover, stereotactic body radiation therapy (SBRT) or transarterial chemoembolization (TACE) could not be performed due to a guarded prognosis. Hence, the patient was started with sorafenib 200 mg orally twice daily and dexamethasone 8 mg daily. Following the goals of care discussion, the patient and his family opt for comfort care. Subsequently, the patient had a cardiac arrest and passed away. The outline of significant events is shown in Figure 3.

## Discussion

HBeAg-negative CHBVI is primarily found in Asia and the Middle East, with a median prevalence of 33% in the Mediterranean, 15% in the Asia Pacific, and 14% in the United States and Northern Europe [9]. About 15-20% of these carriers have raised alanine aminotransferase (ALT) and high viral DNA >100,000 copies/mL [9]. However, limited data is available about HBeAg-negative CHBVI cases in Africa. In our case, we discussed a 40-year-old patient who moved to the United States from Ghana in 2012. The source of acquiring infection was unclear despite the completion of the hepatitis B vaccine series before immigrating to the United States.

After the transition from HBeAg-positive phases (immune tolerant and immune clearance) to HBeAg-negative phases (non-replicative and reactivation), most patients become inactive HBsAg carriers with low viral replication and minimal liver inflammation. However, in some patients, pre-core (with G-for-A substitution at position 1896) and/or basal core promoter (with A-for-T and G-for-A, at positions 1762 and 1764, respectively) mutant variants emerge which reduce or abrogate HBeAg production, leading to HBeAg-negative CHBVI with high viral load (reactivation phase). This results in a high HBV load with an absence of HBeAg leading to extensive liver damage and an increased risk of developing liver cirrhosis and HCC [10]. Gene sequencing is a reliable method for detecting pre-core mutation, but it may not be readily available in all clinical settings. Nonetheless, in routine clinical practice, the diagnosis of CHBVI with the pre-core mutation can be established by the negative HBeAg, a well-recognized marker [11]. Literature reports a significant positive correlation between pre-core/core HBV mutants and rapid progression to HCC [11] due to frequent acute exacerbations characterized by high viral replication, elevated ALT, and accelerated liver necroinflammation [10]. However, our patient is unique; although he was HBeAg negative in 2020 and did not receive treatment during the subsequent two years, the progression of his HCC was slow. It is well-documented that a high HBV-DNA level is associated with recurrence regardless of curative resection of HCC [12]. Although our patient's viral load was consistently low (<10 IU/mL) following the partial hepatic resection, it is unclear whether the patient developed recurrence or mere progression of the undetectable remanent lesion, which could not be definitively answered because of a lack of close monitoring due to lost to follow-up.

Literature highlights disparities in access to care or quality of care among different racial groups for HBV infection. Pham et al. reported that Asians are more likely to receive surveillance testing (e.g., HBV DNA, HBeAg, and liver imaging) within 12 months of CHBVI diagnosis and tend to receive and continue antiviral treatment at 13-24 months compared to White and Black patients [7]. Furthermore, in other studies, the disparity in the provision of care was discussed in relation to the insurance provider. It is unclear why HBV-infected patients with commercial insurance received more frequent testing and treatment than those in Medicare plans. The differences in testing and treatment rates may be related to higher out-of-pocket costs for Medicare patients [7,12]. In 2018, HBV antiviral drugs were classified as tier 5 drugs associated with high out-of-pocket expenses in the Medicare formulary. Since then, costs of antiviral dropped from thousands of dollars per year to below $500 per year for generic tenofovir and entecavir in 2022; however, in many Medicare plans, generic entecavir and tenofovir are still listed as tier 4 drugs [13].

Singal et al. demonstrated in a meta-analysis that HCC surveillance was associated with improved early-stage detection, curative treatment rates, and survival [14]. However, surveillance as per AASLD guidelines in real-life practice is under-utilized, with an adherence rate of less than 20% in American patients with cirrhosis [5,6]. The barriers to HCC surveillance are categorized as patient, provider, and system-related barriers. Among the patient-related barriers, one of the most frequent factors involved was a lack of patient knowledge [15]. One of the most frequent obstacles in the provider-related barrier was a lack of up-to-date knowledge about the disease, its surveillance, and management, as per the AASLD guidelines [15]. In a survey conducted among physicians and residents in training in 2019, 80% indicated that they were not well prepared in training to take care of patients with CHBVI [16]. In another study conducted among 277 primary care physicians (PCP)s affiliated with several major health systems located in a highly prevalent city, authors found nearly half of the providers were unfamiliar with current AASLD guidelines for CHBVI management, and a third of the providers were unaware that antiviral therapy would reduce the risk of liver disease progression [17]. Last but not least, system-related barriers were most commonly related to financial issues, either having no insurance or monetary difficulties that prevented individuals from paying out-of-pocket costs, especially among immigrants, as seen in our case [18]. A study reported that cirrhosis predicts optimal surveillance rates [19]. On the contrary, although our patient had cirrhosis at the time of diagnosis, his lack of insurance coverage and suboptimal understanding of the disease could have hindered his access to adequate care. However, this does not suggest that patients seek care after developing cirrhosis; instead, this statement highlights that patients with CHBVI are more likely to receive comprehensive monitoring and treatment from their physicians as per the surveillance guidelines due to their increased risk of developing HCC.

Electronic health records (EHR) are increasingly used to improve disease screening and immunization in primary care clinics. The clinical decision support tools can be built into EHRs for hepatitis screening and reflex testing for patients with CHBVI, including liver function tests, HBV DNA, HBeAg, and HCC surveillance tests, to assist and improve care and treatment of CHBVI by primary care providers [20]. To improve the disease understanding and adherence to monitoring tests and antiviral therapy by patients, providers should provide language-concordant information and evidence-based and culturally and linguistically appropriate pamphlets about HBV facts and management that can be downloaded from authentic online sources. Patients should also be educated about Government funded programs, utilizing community health centers, and seeking out patient assistance programs for the continuity of care where our patients fell through the crack.

Here, we described one of many unfortunate tales of healthcare disparity in our country. Many patients resort to alternative medicine and herbal supplements due to patient's beliefs, lack of knowledge, and inability to afford healthcare. Predicting how much cancer progression could have been prevented in our patient is difficult. However, the devastating outcomes could have been minimized should the patient be allowed accessible and cost-effective healthcare. Therefore, a comprehensive national HBV elimination strategy is urgently needed to reach the global and the United States goals to eliminate hepatitis B as a public health threat by 2030, including initiatives to address patient and provider-related barriers.

## Conclusions

Patients with CHBVI require regular monitoring of their viral load, liver function tests, and imaging studies, even if they have achieved a sustained virological response to antiviral therapy. Adhering to antiviral treatment is critical in preventing disease progression. However, vulnerable and marginalized populations, such as low-income, racial and ethnic minorities, and rural communities, face significant healthcare disparities, worsening health outcomes, including higher morbidity and mortality rates. Therefore, healthcare providers, policymakers, and public health professionals must address these disparities and strive to achieve health equity.

## References

[1] Meireles LC, Marinho RT, Van Damme P (2015). Three decades of hepatitis B control with vaccination. World J Hepatol.

[2] El-Serag HB (2012). Epidemiology of viral hepatitis and hepatocellular carcinoma. Gastroenterology.

[3] Yim HJ, Lok AS (2006). Natural history of chronic hepatitis B virus infection: what we knew in 1981 and what we know in 2005. Hepatology.

[4] Terrault NA, Lok AS, McMahon BJ (2018). Update on prevention, diagnosis, and treatment of chronic hepatitis B: AASLD 2018 hepatitis B guidance. Hepatology.

[5] Davila JA, Morgan RO, Richardson PA, Du XL, McGlynn KA, El-Serag HB (2010). Use of surveillance for hepatocellular carcinoma among patients with cirrhosis in the United States. Hepatology.

[6] Singal AG, Yopp A, S Skinner C, Packer M, Lee WM, Tiro JA (2012). Utilization of hepatocellular carcinoma surveillance among American patients: a systematic review. J Gen Intern Med.

[7] Pham TT, Toy M, Hutton D (2023). Gaps and disparities in chronic hepatitis B monitoring and treatment in the United States, 2016-2019. Med Care.

[8] Cohen C, Holmberg SD, McMahon BJ (2011). Is chronic hepatitis B being undertreated in the United States?. J Viral Hepat.

[9] Funk ML, Rosenberg DM, Lok AS (2002). World-wide epidemiology of HBeAg-negative chronic hepatitis B and associated precore and core promoter variants. J Viral Hepat.

[10] Alexopoulou A, Karayiannis P (2014). HBeAg negative variants and their role in the natural history of chronic hepatitis B virus infection. World J Gastroenterol.

[11] Akahane Y, Yamanaka T, Suzuki H (1990). Chronic active hepatitis with hepatitis B virus DNA and antibody against e antigen in the serum. Disturbed synthesis and secretion of e antigen from hepatocytes due to a point mutation in the precore region. Gastroenterology.

[12] Wong RJ, Jain MK, Therapondos G, Niu B, Kshirsagar O, Thamer M (2022). Low rates of hepatitis B virus treatment among treatment-eligible patients in safety-net health systems. J Clin Gastroenterol.

[13] (2022). Blue Cross Medicare Advantage Flex (PPO). https://www.bcbsil.com/medicare/pdf/2022/mapd-formulary-ppo-flex-il-2022.pdf.

[14] Singal AG, Pillai A, Tiro J (2014). Early detection, curative treatment, and survival rates for hepatocellular carcinoma surveillance in patients with cirrhosis: a meta-analysis. PLoS Med.

[15] Ispas S, So S, Toy M (2019). Barriers to disease monitoring and liver cancer surveillance among patients with chronic hepatitis B in the United States. J Community Health.

[16] Chao SD, Wang BM, Chang ET, Ma L, So SK (2015). Medical training fails to prepare providers to care for patients with chronic hepatitis B infection. World J Gastroenterol.

[17] Mukhtar NA, Kathpalia P, Hilton JF (2017). Provider, patient, and practice factors shape hepatitis B prevention and management by primary care providers. J Clin Gastroenterol.

[18] Burman BE, Mukhtar NA, Toy BC (2014). Hepatitis B management in vulnerable populations: gaps in disease monitoring and opportunities for improved care. Dig Dis Sci.

[19] Wang C, Chen V, Vu V (2016). Poor adherence and low persistency rates for hepatocellular carcinoma surveillance in patients with chronic hepatitis B. Medicine (Baltimore).

[20] Konerman MA, Thomson M, Gray K, Moore M, Choxi H, Seif E, Lok AS (2017). Impact of an electronic health record alert in primary care on increasing hepatitis c screening and curative treatment for baby boomers. Hepatology.

